# The Microbiome Characterization of Edible Visceral Organs and Fresh Meat During Production in a Pig Processing Facility in Thailand

**DOI:** 10.3390/pathogens14050475

**Published:** 2025-05-14

**Authors:** Jutamat Klinsoda, Alongkot Boonsoongnern, Narut Thanantong, Tanyanant Kaminsonsakul, Khemmapas Treesuwan, Sudsai Trevanich, Barbara U. Metzler-Zebeli

**Affiliations:** 1 Institute of Food Research and Product Development, University of Kasetsart, Bangkok 10900, Thailand; ifrjmk@ku.ac.th (J.K.); khemmapas.tr@ku.th (K.T.); 2 Department of Farm Resources and Production Medicine, Faculty of Veterinary Medicine, University of Kasetsart Kamphaeng Saen, Nakhon Pathom 73140, Thailand; fvetakb@ku.ac.th (A.B.); narut.t@ku.th (N.T.); tanyanant.k@ku.th (T.K.); 3 Department of Food Science and Technology, Faculty of Agro-Industry, University of Kasetsart, Bangkok 10900, Thailand; sudsai.t@ku.th; 4 Centre for Veterinary Systems Transformation and Sustainability, Clinical Department for Farm Animals and Food System Science, University of Veterinary Medicine Vienna, 1210 Vienna, Austria

**Keywords:** microbiome, pig, visceral organs, slaughterhouse, pork, bacterial transmission

## Abstract

Besides meat, pig organs are traditionally consumed in Asia. However, they can be a source of food poisoning. Less is known about the microbiome associated with different organ meats and the inter-animal variation in the microbiomes of organs. The aim of this pilot study was to characterize and compare the bacterial composition in fresh pig meat and organs (i.e., tonsils, lungs, and spleen) and blood from several carcasses using 16S rRNA amplicon sequencing as a screening method. We also investigated how closely the bacterial composition of the meat and organ samples was related to the gut bacterial community and the bacterial communities on the hands of the workers at different positions during meat processing. Meat, organ, blood, and gut (cecum and feces) samples were collected from 12 carcasses in two batches (*n* = 6/batch), along with swab samples (*n* = 4/batch) from the hands of the workers at different positions along the processing chain, from which DNA was extracted. The results for the bacterial diversity showed that each sample type (meat, organ, and blood) comprised a unique taxonomic composition (*p* < 0.05). Moreover, the data confirmed great inter-animal and batch variation for the meat, organs, and blood, which is helpful information for implementing strategies to enhance hygiene measures at pig farms and slaughterhouses, and hence food safety and quality. The genera associated with food safety and spoilage, such as *Anoxybacillus*, *Acinetobacter*, *Pseudomonas*, *Campylobacter*, and *Streptococcus*, were also different between the meat, organs, and blood. The bacterial communities in the gut samples distinctly clustered from communities in the pig organs and meat, whereas some overlaps in community clusters between lung, meat, and hand samples existed. This study demonstrates that the spleen, tonsils, and lungs contained more bacterial genera that comprise pathogenic strains than meat cuts, supporting the need to monitor their microbiome composition as potential contamination sources for food safety and spoilage reasons.

## 1. Introduction

The consumption of pork is traditionally high in certain Asian countries, like Thailand [1]. Besides the meat, visceral pig organs (i.e., liver, lung, heart, intestine, and blood) are traditionally consumed, and food poisoning has often been related to raw meat and organ consumption in these countries [2,3]. Investigating the factors influencing microbial ecology in meat products is complex because each carcass may come with its own microbial community [4,5]. In this respect, different meat cuts have been shown to vary in their microbial composition [4]. Relatively little is known about the microbial ecology of edible organs and blood, and whether the diversity of the microbiome is similar among different organ types. Moreover, little is known as to whether the handling of visceral organs similarly affects the microbiota in the processing area. During the slaughter process, contamination can occur from the porcine gut during evisceration, from airborne microorganisms, and during packaging, which are transferred via the hands of the workers and processing tools [4,5]. In general, the gut microbiota is thought to be the most important source of carcass contamination. However, visceral organs that represent immune organs, like tonsils, may act as a reservoir for zoonotic agents [6], which can be spread onto the pork carcass during cutting and meat inspection at the processing plant [6]. Consequently, the microbes present in lymphatic tissues can become a health risk for the consumer if organs are consumed raw or half-cooked [7,8]. These microbes may impact the microbial characteristics of fresh pork [4] as they can speed up meat spoilage, leading to economic losses and food waste [9]. In order to improve the preventive and response measures for food safety and quality, a better understanding of the microbial ecology in pork products, including visceral organs and blood, is needed. It can be assumed that the microbiome composition in visceral organs, such as lungs and tonsils, is related to the health status of the individual animal or herd. Several studies have confirmed that microbiome profiling of the lung [10] and tonsil microbiome [11] reflects the animals’ health status and performance. Therefore, information about the microbial community in visceral organs may be useful for the implementation of on-farm strategies to improve the health status of the animals in the future.

Recent research supports the usefulness of high-throughput 16S rRNA amplicon sequencing for microbial screening purposes of meat samples [5,12] and contact surfaces in pork processing facilities [13,14]. Therefore, we applied this technique in the present pilot study to characterize and compare the bacterial composition of fresh pig meat and organs (i.e., tonsils, lungs, and spleen) and blood from several carcasses. We also investigated how closely the bacterial composition of the meat and organ samples is related to the gut bacterial community and the bacterial communities on the hands of workers at different positions during meat processing. This study was based on the hypothesis that, due to their role as immune organs, more genera comprising pathogenic and meat-spoiling taxa would be found in visceral organs, such as lungs and tonsils, compared to in the meat and blood.

## 2. Materials and Methods

### 2.1. Ethical Approval

The animal experiment was conducted in accordance with the letter of approval for animal care and use for scientific research, Kasetsart University (ID ACKU65-ETC-001), under the ethical review board of the Office of the National Research Council of Thailand.

All swab testing from humans was conducted in accordance with the international guidelines for human research protection under the Declaration of Helsinki, the Belmont Report, the CIOMS Guideline, and the International Conference on Harmonization in Good Clinical Practice (ICH-GCP). The Certificate of Approval (COA No. COA65/022) was given by the Kasetsart University Research Ethics Committee. The informed consent forms for the swab test were obtained from all workers.

### 2.2. Animals and Sampling

Healthy male pigs (Large white × Land race × Duroc) from a medium-sized farm in the central region of Thailand at slaughter weight (BW = 105–110 kg) were selected from two fattening periods with a 3-month interval (n = 6/batch). The procedures at the slaughterhouse were similar between the two batches. At the farm, pigs received an ear tag with an identification number, which was used to follow the samples at the slaughterhouse and in the carcass processing chain.

### 2.3. Sample Collection

Pigs were processed at one slaughterhouse in Nakhon Pathom, Thailand. The evisceration and sampling of each pig were sequentially done by the workers at the slaughterhouse. The whole gut was removed from the carcass and placed in a sterile plastic bag. The tonsils of the pigs were cut from the heads of the pigs under sterile conditions. Blood was taken from the heart of each dead pig, collected in 15 mL EDTA blood collection tubes (Greiner Bio-One Ltd., Chonburi, Thailand). The tissue pieces from the tonsils, lungs, spleen, and the left hindleg meat of each pig were placed into one plastic bag per tissue per animal before transportation. After finishing work, the workers from the different positions (no. 1–2: gut and tissue remover, no. 3–4: carcass trimmer, no. 5–6: organ processor, and no. 7–8: product packer) were called to do a hand swab test (*n* = 4 samples/replicate; workers no. 1, 3, 5, and 7 in batch 1 and workers no. 2, 4, 6, and 8 in batch 2). The hands of the workers were sampled by 3M™ Swab-Sampler with Neutralizing Buffer (3M Health Care, Eden Prairie, MN, USA), which were stored on ice during transport. The processing time between stunning and the completion of sample collection was 30 min.

Samples were transferred on ice to the laboratory for subsampling. The small and large intestines were carefully dissected from the mesentery, and clamps were used to prevent the mixing of digesta between the intestinal segments. The samples of the lungs, tonsils, spleen, and meat were subsampled by cutting them aseptically into smaller pieces, placing them into cryotubes, and snap-freezing them in liquid nitrogen. The cecal digesta were mixed, and the aliquots were placed into cryotubes. The cecal mucosa were cleaned with sterile PBS, cut into small pieces, and snap-frozen in liquid nitrogen. The fecal samples were collected from the rectal part of the gut, placed into cryotubes, and were snap frozen in liquid nitrogen. The gut, organ, meat, and fecal samples were stored at −80 °C until microbial analysis. From the blood samples, DNA was immediately extracted. The remaining blood was stored at −0 °C.

### 2.4. DNA Extraction

The extraction of DNA from the blood, feces, cecal digesta, cecum mucosa, and pork organs, as well as the bacterial cells from the enrichment culture of the hand swabs, was performed using the DNeasy Blood and Tissue Kit (Qiagen, Hilden, Germany), according to the manufacturer’s instructions. The DNA concentration was measured with a Nanodrop (Thermo Fisher Scientific Inc., Waltham, MA, USA). Some samples of the meat (n = 4), lungs (n = 2), spleen (n = 1), feces (n = 1), and blood (n = 2) were excluded from sequencing due to failure in library construction. Possible reasons were low microbial biomass and the presence of polymerase chain reaction (PCR) inhibitors.

### 2.5. 16S rRNA Amplicon Sequencing and Bioinformatics

The DNA samples with A260/A280 absorbance ratios of 1.8–2.0 were submitted to Novogene (Beijing, China) for 16S rRNA amplicon sequencing using Illumina NovaSeq 6000 (Illumina, San Diego, CA, USA). The V3-V4 region was amplified (341F: CCTAYGGGRBGCASCAG and 806R: GGACTACNNGGGTATCTAAT). The TruSeq^®^ DNA PCR-Free Sample Preparation Kit (Illumina, USA) was used for library construction. The PCR products were then purified using the Qiagen Gel Extraction Kit (Qiagen, Hilden, Germany). The prepared libraries were sequenced to an expected sequencing depth of 100,000 paired-end reads. After sequencing, the paired-end reads were assigned to samples based on their unique barcode and truncated by cutting off the barcode and primer sequence. Negative controls (n = 2) were included to control for potential DNA contamination. The quality filtering of the raw reads was performed under specific filtering conditions to obtain clean reads, using Novogene.

The raw sequencing reads (Fastq files) were performed using the Divisive Amplicon Denoising Algorithm 2 (DADA2) package (version 1.26.0) in R studio, similar to [15]. The raw amplicon sequencing data were filtered, and chimeras were removed using the “removeBimeraDenovo” command. After the de-replication of the filtered data, error rates were estimated, and amplicon sequence variants were inferred at a 99% identity. The taxonomy was assigned using the SILVA database (version 138.1), with a dissimilarity threshold of 3% as a reference template. The α-diversity (Shannon and Simpson) and species richness (observed features) were assessed using the R package ‘phyloseq’ (version 1.42.0). The β-diversity analysis (Bray–Curtis distance) was assessed using the ‘adonis2’ function (PERMANOVA) in the R package ‘vegan’ (version 2.6.4) [16]. The clustering of genera from different sample locations, the gut (feces or cecal digesta and mucosa), pork, organs, and hands of the workers, was visualized using two-dimensional nonmetric multidimensional scaling (NMDS) ordination plots obtained with the ‘metaMDS’ function in the vegan R package.

### 2.6. Statistics

The raw read counts from each sample were collapsed and compositionally normalized such that each sample summed to 1. The relative abundances at the respective taxonomic ranks were analyzed. The data were tested for normal distribution by using the Shapiro–Wilk test in R (version 4.4.3). Non-normally distributed data were log-transformed for the ANOVA. The α-diversity, the relative abundances of bacteria in different datasets, were subjected to ANOVA with a mixed model, using the lme4 package in R. The fixed effects included the sample type and pig. The random effect was the replicate batch. The experimental unit was the respective sample from each pig. The data were expressed as least-squares means ± standard error of the mean (SEM), which were created by the ‘lsmeans’ package in R. The differences between least-squares means were tested using Tukey’s HSD test. A significant difference was considered at *p* < 0.05. The heatmapping and clustering of the genera and samples were performed using the pheatmap R package.

## 3. Results

### 3.1. Bacterial Community Structure

**Figure 1:**
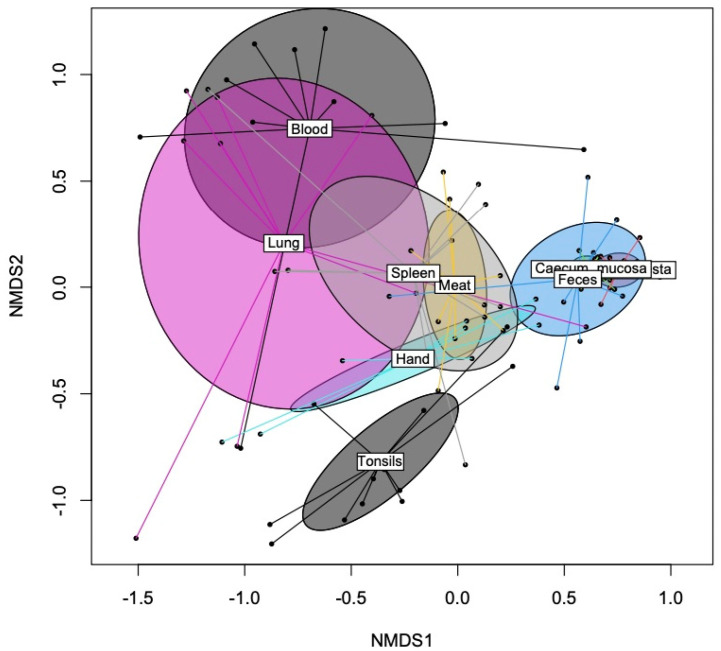
Non-metric multidimensional scaling (NMDS) ordination plot, based on Bray-Curtis dissimilarities for bacterial microbiomes among sample types (i.e., feces, blood, lungs, tonsil, cecum, spleen, meat cut, and carcass handlers’ hand).

The 16S rRNA amplicon sequencing identified a total of 716 genera across the sample types. Bacterial species richness and diversity were largely different among sample types (*p* < 0.001; Table 1). The cecal mucosa had the highest bacterial species richness (observed features), whereas blood and the swab samples from the hands of the workers had the lowest (*p* < 0.001). The alpha diversity (Shannon and Simpson) of the bacterial community in the cecal mucosa, cecal digesta, and feces was higher than that in the blood, lung, spleen, and hand swab samples (only Shannon). The PERMANOVA (Bray–Curtis distance) confirmed distinct microbial communities among the sample types (*p* = 0.001; Table S1). The NMDS plot illustrated that the bacterial communities in the gut samples (feces, cecal digesta, and mucosa) were distinct from those in the meat, organ, and blood samples, as well as from the hand swab samples (Bray–Curtis distance; Figure 1). The bacterial communities in the spleen, meat, and lungs overlapped with those of the swab samples from the hands of the workers. The bacterial communities in the blood samples, in turn, clustered distinctly from the meat, gut, tonsil, and hand samples but overlapped with the communities in the lung and spleen samples.

### 3.2. Bacterial Composition of Blood, Visceral Organs, and Meat

**Figure 2:**
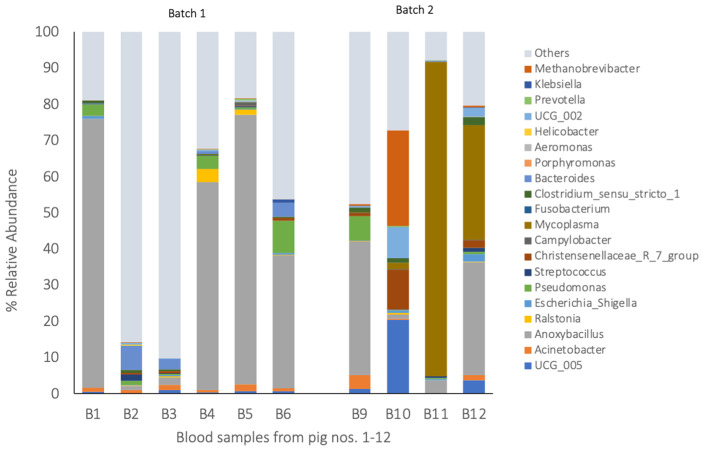
The 20 most abundant bacterial taxa in the blood samples (the relative abundance in % of all the reads). The bacterial communities at the genus level with a relative abundance > 1.4%. Blood: B1–B12 stands for the blood samples from pigs nos. 1 to 12.

**Figure 3:**
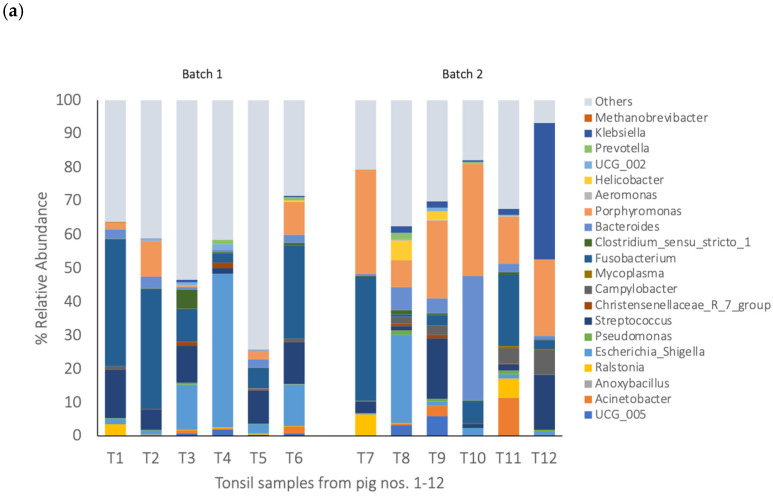

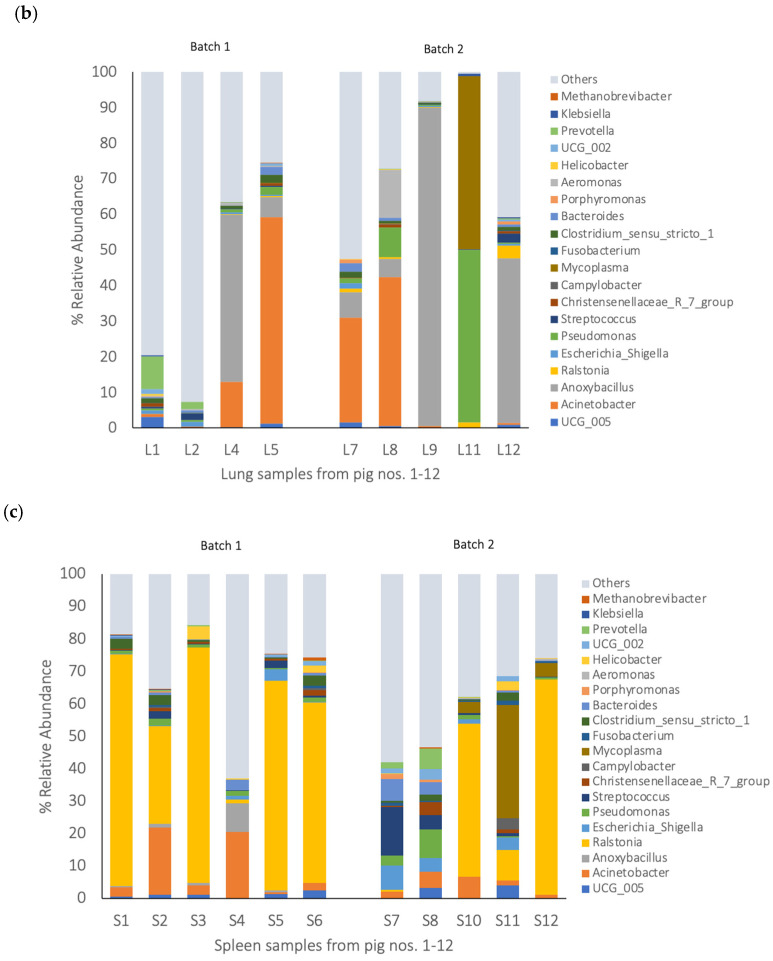
The 20 most abundant bacterial taxa in the pig organs (the relative abundance in % of all the reads). The bacterial communities at the genus level with a relative abundance > 1.4%. (**a**) Tonsils: T1–T12 stands for the tonsil samples from pigs nos. 1 to 12. (**b**) Lungs: L1–L12 stands for the lung samples from pigs nos. 1 to 12. (**c**) Spleens: S1–S12 stands for the spleen samples from pigs nos. 1 to 12.

**Figure 4:**
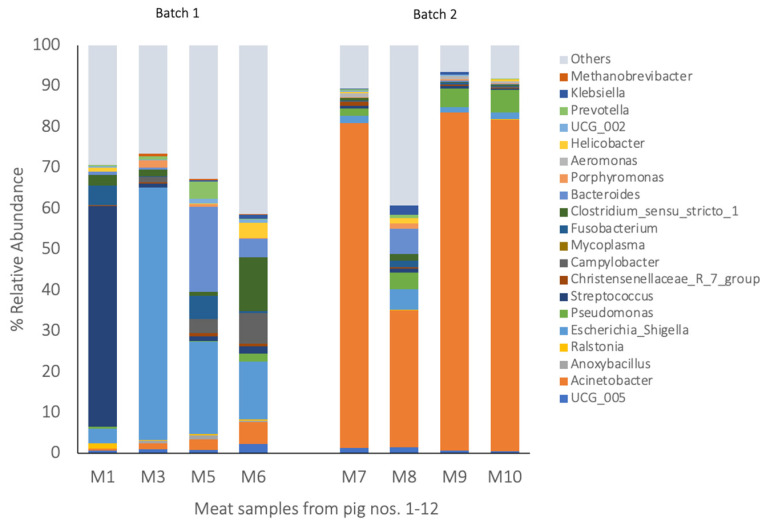
The 20 most abundant bacterial taxa in the meat (the relative abundance in % of all the reads). The bacterial communities at the genus level with a relative abundance > 1.4%. Meat cut: M1–M12 stands for meat samples from pigs nos. 1 to 12.

**Figure 5:**
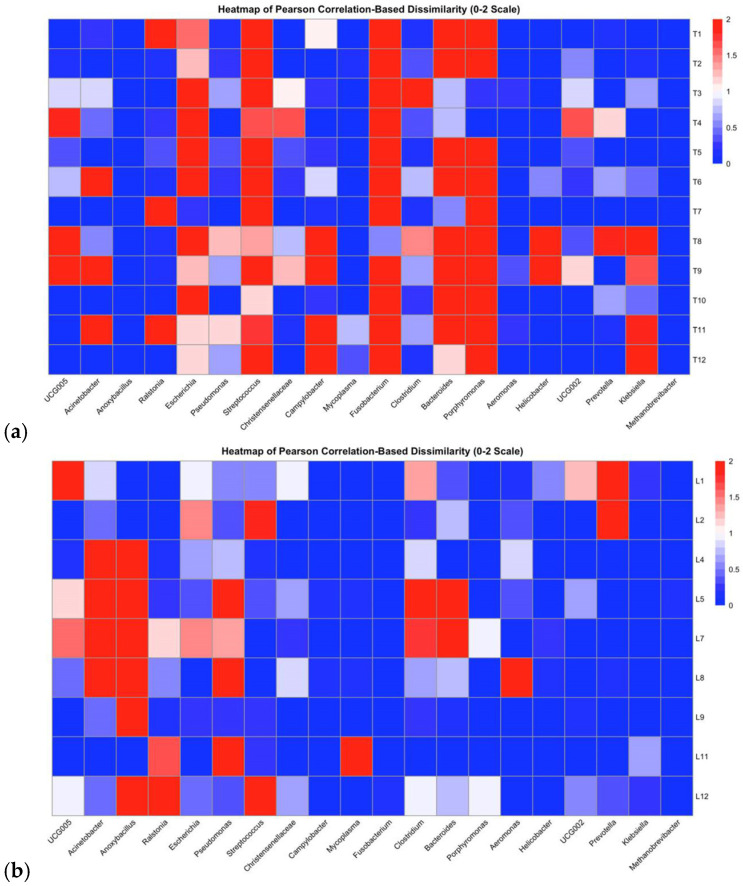

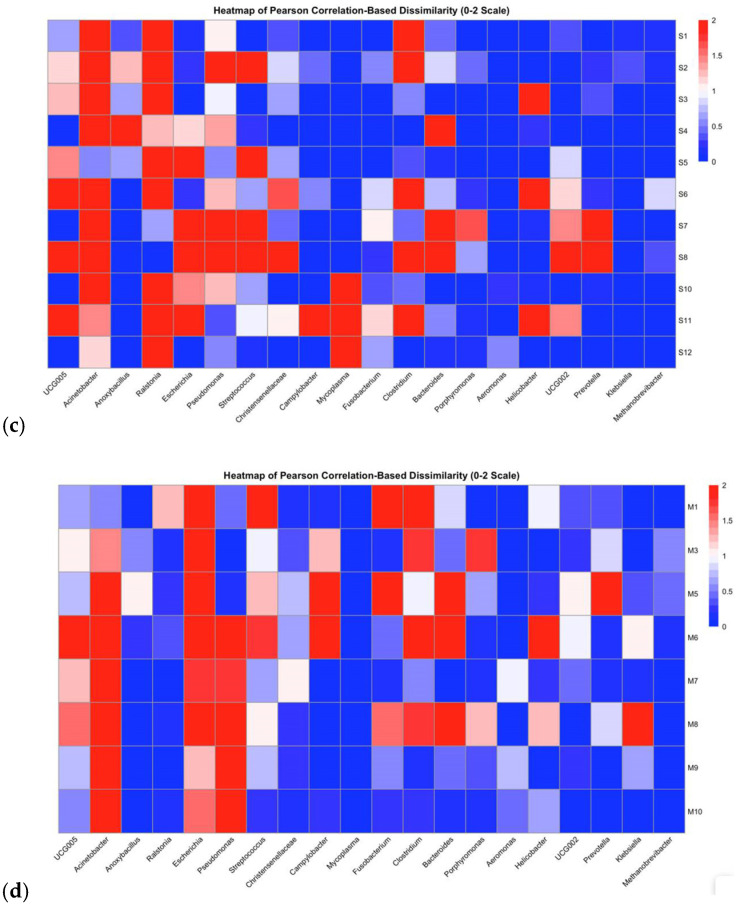
Heatmap illustrating relative abundance of 20 most abundant bacterial genera (relative abundances) in pig organs: (**a**) tonsils, (**b**) lungs, (**c**) spleen, and (**d**) meat. Red and blue colors are scaled with dissimilarity ranging from 0 (r = 1; positive correlation) to 2 (r = −1; negative correlation). Tonsils: T1–T12 stands for tonsil samples from pig nos. 1 to tonsil samples from pig nos. 12. Lungs: L1–L12 stands for lung samples from pig nos. 1 to lung samples from pig nos. 12. Spleens: S1–S12 stands for spleen samples from pig nos. 1 to spleen samples from pig nos. 12. Meat cut: M1–M12 stands for meat samples from pig nos. 1 to meat samples from pig nos. 12.

The blood, tonsils, spleen, lungs, and meat each comprised a unique bacterial composition (Figure 2, Figure 3 and Figure 4). A Pearson’s correlation-based dissimilarities for the visceral organs and meat showed great inter-animal variations in the bacterial composition between individual pigs in both batches (Figure 5). When comparing the relative bacterial abundances for the blood, visceral organs, and meat, the genus *Anoxybacillus*, which is related to meat spoilage, dominated in the blood of four pigs from batch 1 (pigs nos. 1, 4, 5, and 6) and in the blood of two pigs from batch 2 (pigs nos. 9 and 12), with an average relative abundance of 32% (Figure 2; Table S2). *Mycoplasma* was found in high relative abundance in the blood of pigs nos. 11 and 12. In batch 1, the blood samples from pigs nos. 2 and 3 were differently composed than the other three samples. The same applied to the blood sample from pig no. 10 in batch 2, whose composition differed from the other three samples. The relative bacterial composition in the tonsil and lung samples showed great inter-animal and inter-batch variation (Figure 3a,b). For batch 1, several samples contained a high relative abundance of *Streptococcus* (pigs nos. 1, 2, 3, 4, and 6; Figure 3a), *Fusobacterium* (pigs nos. 1, 2, 3, 5, and 6), and *Escherichia* (pigs nos. 3, 4, and 6). For batch 2, there was a general trend that the tonsil samples from all the pigs contained a higher relative abundance of *Porphyromonas*, which was not detected in batch 1. Regarding the lung samples (Figure 3b), pigs nos. 4, 5, 7, and 8 comprised higher relative abundances of *Acinetobacter*, a meat spoilage bacterium, whereas the microbiota in the lung sample of pig no. 9 was almost completely composed of *Anoxybacillus*. Also, pigs nos. 4 and 12 had a high abundance of *Anoxybacillus* in their lungs. Pig no. 11 contained mainly *Pseudomonas* and *Mycoplasma* (approximately a 45% relative abundance of all reads for each of the two taxa) in their lungs. *Pseudomonas* is a common cause of meat spoilage, whereas *Mycoplasma* has zoonotic potential. Seven out of eleven spleen samples comprised *Ralstonia* as the dominating taxon (Figure 3c). The spleen samples of pigs nos. 7 and 8 from batch 2 were different in their composition compared to the other samples. Moreover, pig no. 11 contained a relatively high abundance of *Mycoplasma* (approximately 30% of all reads). Regarding the meat samples (Figure 4), in addition to the inter-animal differences, the bacterial composition was very different in batches 1 and 2. In batch 1, the meat sample from pig no. 1 was dominated by *Streptococcus*, whereas those from the other pigs were characterized by a high abundance of *Escherichia*, *Bacteroides,* and *Clostridium sensu stricto*-1. In batch 2, *Acinetobacter* was the most dominant genus in meat samples across all pigs. The heatmaps of the Pearson’s correlation-based dissimilarities support the inter-animal and inter-batch variations (Figure 5).

### 3.3. Bacterial Composition of Gut Samples

**Figure 6:**
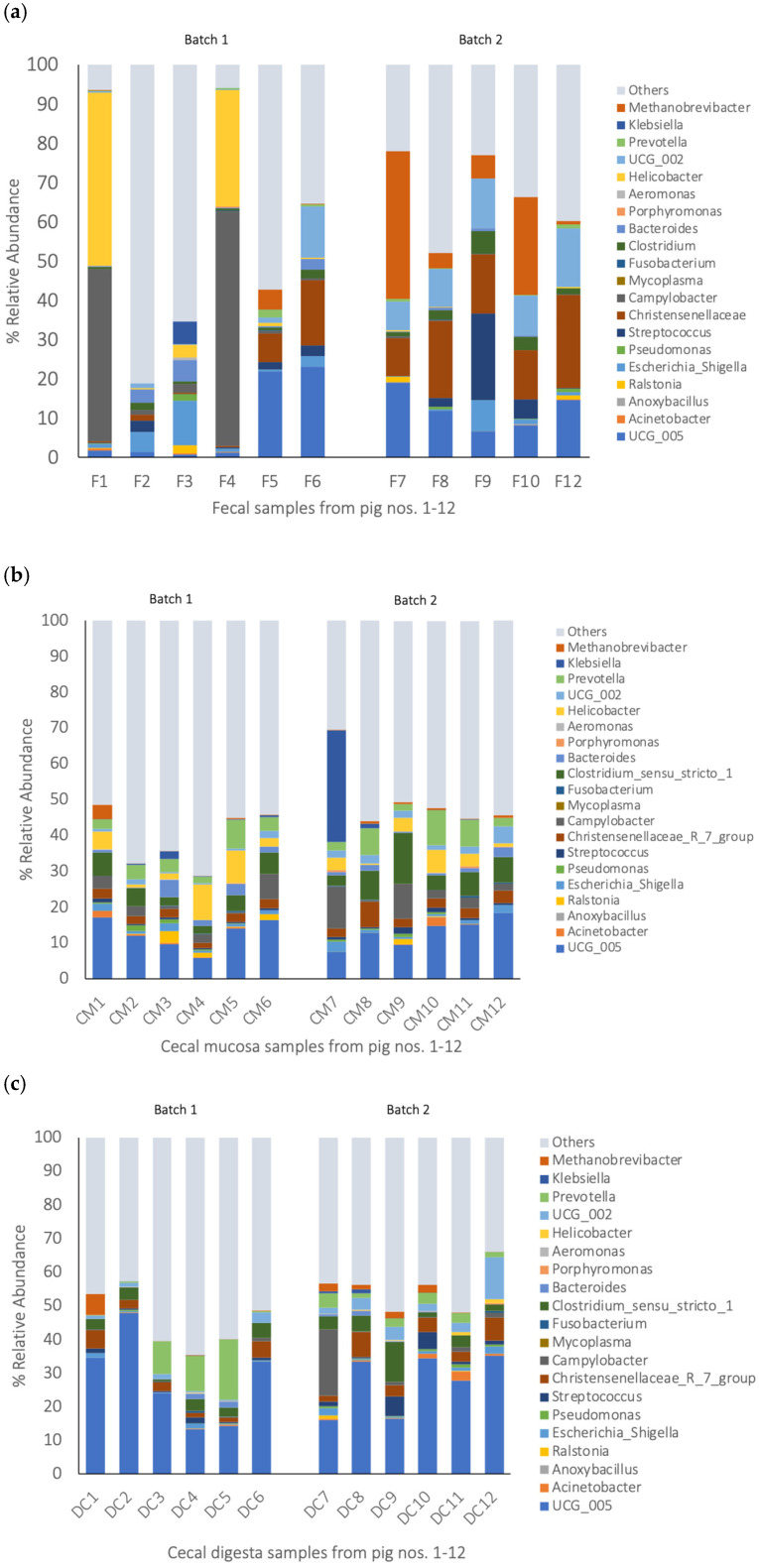
The 20 most abundant bacterial genera in the gut samples of the pigs (the relative abundance in % of all the reads). The bacterial community at the genus level with a relative abundance > 1.4%. (**a**) Feces: F1–F12 stands for the fecal samples from pigs nos. 1 to 12. (**b**) Cecal mucosa: CM1–CM12 stands for the cecal mucosa samples from pigs nos. 1 to 12. (**c**) Cecal digesta: DC1–DC12 stands for the cecal digesta samples from pigs nos. 1 to 12.

Regarding the gut samples, the fecal samples showed a greater inter-animal variation than the cecal digesta and mucosa samples (Figure 6). It is worth highlighting that the majority of the bacteria in the fecal samples from pigs nos. 1 and 4 were *Helicobacter* and *Campylobacter*. The most abundant genus in the cecal mucosa was an unclassified *Oscillospiraceae* genus (UGG-005) with a 12.8% relative abundance (Figure 6b; Table S2), followed by *Prevotella* and *Lactobacillus*, and genera which comprise opportunistic pathogens, such as *Clostridium sensu stricto*-1 (5.8%), *Helicobacter* (3.9%), and *Campylobacter* (3.8%). In the cecal digesta, an unclassified *Oscillospiraceae* genus (UCG-005) was predominant at a 27.5% relative abundance in all the pigs (Figure 6c; Table S2). Genera comprising opportunistic pathogens, such as *Clostridium sensu stricto*-1, *Campylobacter,* and *Streptococcus* (both potential zoonotic agents), were present at a relative abundance of 3.8, 2.1, and 1.6%, respectively.

### 3.4. Bacterial Composition of Hand Swab Samples

**Figure 7:**
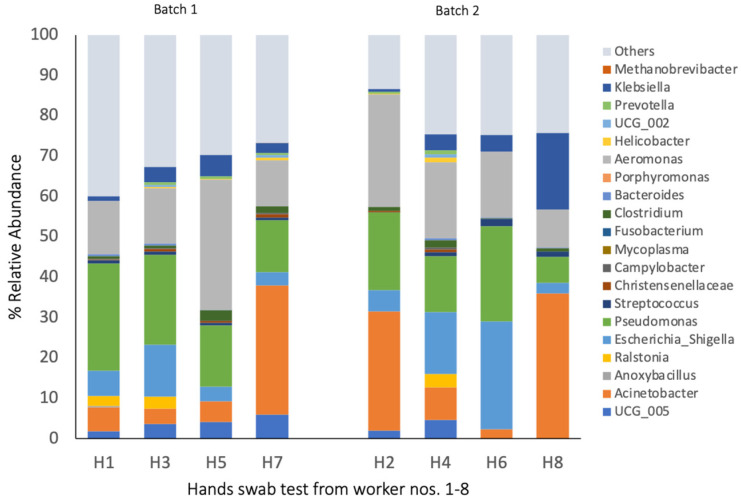
The 20 most abundant bacterial taxa in the swab samples from the hands of the workers (the relative abundance in % of all the reads). The bacterial communities at the genus level with a relative abundance > 1.4%. The swab samples from the hands were enriched for 24 h before DNA extraction. H1–H2 stand for the hands of the gut and tissue remover workers; H3–H4 stand for the hands of the carcass trimmer workers; H5–H6 stand for the hands of the organ processor workers; and H7–H8 stand for the hands of the product packer workers. The 1st replicate from workers nos. 1, 3, 5, and 7, and the 2nd replicate from workers nos. 2, 4, 6, and 8.

The bacterial composition of the hands of the workers differed depending on their position in the processing chain and between the two batches. *Pseudomonas* and *Aeromonas* were dominating taxa present on the hands in both batches (Figure 7; Table S2), whereas *Escherichia* was abundant at 9.5% (*p* < 0.001). In batch 1, *Pseudomonas* and *Aeromonas* were highly abundant on the hands of worker no. 5, who processed the organs (32%), whereas *Acinetobacter* was highly present on the hands of worker no. 7, who was a product packer (32% relative abundance). In batch 2, *Acinetobacter* was highly abundant on the hands of two workers [no. 2 (gut and tissue remover) and 8 (product packer)] at a 29 and 35% relative abundance, respectively. Worker no. 8 (product packer) also had a high abundance of *Klebsiella* with a 18.8% relative abundance on his hands 8. Across the batches, the product packers (nos. 7 and 8) had relatively similar bacterial communities on their hands (Figure 4).

## 4. Discussion

In Thailand and Asia, the consumption of raw pig meat and organs is part of the traditional food culture, which is associated with a higher risk of food poisoning in these (sub-)tropical regions. However, little is known about the bacteria present in visceral organs, such as the lungs, spleen, and tonsils, as well as in the blood, and thus, little is known about their potential as a risk factor for microbial contamination of pork in the slaughterhouse. Microbiome analysis, using 16S rRNA amplicon sequencing, revealed highly diverse yet distinct bacterial communities in the lungs, spleen, tonsils, and blood, which differed (except for spleen) from those in the meat, which is important information given the final usage and preparation of the meat and organs specifically. Although the slaughter pigs came from the same farm, the results not only showed differences between the batches for the meat, blood, and organs, but also a large animal-to-animal variation in the bacterial compositions, as demonstrated by the heatmaps for the Pearson’s correlation-based dissimilarities. This variation can probably be explained by the different microbial exposures at the farm and the slaughterhouse between the two batches. In addition to contamination during evisceration and handling of the carcasses and organs by the workers, the reason for the differences in the bacterial microbiomes between the two batches may be due to changes in the bacterial environment at the slaughterhouse from batch 1 to 2 [4,12]. Therefore, it is recommended to collect environmental and fecal samples at the farm before transport and more environmental samples (biomapping) at the slaughterhouse to identify sources of contamination and/or microbial variation in meat and organ products in future studies. The gut samples can provide an idea about the extent to which the farm may have influenced the microbiome of the animals between the batches [17,18,19]. While the cecal samples (mucosa and digesta) were relatively similar between batches, the fecal samples support that changes in the environmental microbes at the farm may have played a role [19]. The present observation about animal-to-animal and batch-to-batch variation may be helpful for risk assessment regarding food safety and the implementation of intervention strategies on farms to improve the health of individual animals before slaughter. Furthermore, the results confirmed our hypothesis that the spleen, tonsils, and lungs contain more genera comprising zoonotic pathogens than the meat pieces, supporting that their raw consumption may not be safe. In addition, certain blood samples contained *Mycoplasma*, which shows that blood needs to be handled as carefully as organs in terms of food safety.

The diverse bacterial communities on the hands of the workers and, as indicated by the overlapping clusters in the NMDS plot, their similarity to the bacterial communities in the meat, spleen, and lung samples highlights the importance of good hygienic practice to avoid the unnecessary contamination of the carcass and organs during processing and packaging [4,5]. The different workers at the various positions in the carcass processing chain had different dominant genera on their hands, highlighting the importance of good hygienic practice to minimize the spread of meat spoilage and disease agents. Our results demonstrate the importance of enhanced hygiene measures to minimize the microbial contamination of the meat cuts and as self-protection for the workers. The similarities between the fecal and cecal communities with the other sample types were low, as indicated by the clustering in the NMDS plot. Nevertheless, when comparing the taxonomic composition, the high relative abundance of *Escherichia* in the meat (pigs nos. 3, 5, and 6) of batch 1 may have been due to the contamination of the carcass during evisceration. In order to reduce any potential contamination of the carcass and inner organs, it may be recommended to use a plastic bag to seal the rectum during the evisceration of the individual carcasses [20]. In fact, the procedure of sealing the rectum should be more emphasized at pig processing facilities to reduce the contamination risk.

The gut, as the major site of bacterial colonization of the body, is known to comprise a highly diverse bacterial community [21]. Correspondingly, the present gut samples (i.e., feces, cecal digesta, and mucosa) were highly diverse. However, the current data also showed that the microbial communities of the tonsil and meat samples carried a microbiome that was as diverse (Shannon and Simpson) as the gut samples. As the three bacterial communities clustered apart in the NMDS plot, the high diversity of the meat and tonsil samples cannot be solely explained by contamination from the gut. For the tonsils, the higher diversity was probably associated with their role as an immune organ in the body. Their main role is to filter out ingested or inhaled microbes, preventing them from reaching systemic circulation [22]. Therefore, the bacterial community found in the tonsils potentially reflected the microbial environment of the pig barn. Consequently, the abundances of *Escherichia*, *Fusobacterium*, *Bacteroides*, *Porphyromonas,* and *Klebsiella* in tonsils of some of the pigs may be explained by the oral uptake of fecal matter in the barn and not necessarily by contamination during carcass processing at the slaughterhouse. Differential abundance analysis indicated that the majority of the bacteria present in the meat samples belonged to a few genera, such as *Streptococcus*, *Escherichia*, *Bacteroides*, *Campylobacter,* and *Clostridium* sensu stricto-1 in batch 1 and *Acinetobacter*, *Pseudomonas*, *Bacteroides,* and *Prevotella* in batch 2. This means that the observed high diversity of the bacterial community in the meat samples was mainly due to low-abundant taxa. Despite their low abundance at the time of slaughter, there is a potential for outgrowth during the shelf life of the meat [4]. The high abundance of key spoilage bacteria in the meat, i.e., *Acinetobacter* and *Pseudomonas* [23], at the time of carcass processing is particularly relevant for the shelf life of the meat. Higher abundances of *Escherichia*, *Campylobacter,* and *Clostridium* sensu stricto-1, in turn, are of great relevance from the perspective of food safety [24]. *Campylobacter* is a common gut inhabitant in pigs. A high prevalence of *Campylobacter* in meat, however, is a food safety concern as it can cause human illness. Accordingly, it would not have been advisable to consume meat from batch 1 in a raw form. Moreover, the genus *Streptococcus* was present in almost all the tonsil samples and was the dominating taxon in the meat sample from pig no. 1. Due to the high prevalence of *Streptococcus suis* in Asia and its zoonotic potential [25], careful removal of the tonsils from the carcass is recommended to minimize the spread of *Streptococcus* to the meat [3,26,27]. Accordingly, Liang et al. [28] and Wongnak et al. [29] reported that the tonsils were the origin of *Streptococcus* contamination in pork meat.

Although the bacterial species richness was lowest for the blood samples, its bacterial diversity (Shannon) was similar to the other organs, such as the lungs and spleen. As the blood sample was collected directly from the heart of the animal, contamination from the environment can be mostly excluded as a source for the bacteria. Therefore, the bacterial ecology observed for the blood was mainly associated with the health status of the individual pig. The mucosal immune system of the respiratory tract and lymphatic system filters and accumulates the invading pathogenic microorganisms as a defense mechanism [30]. The lungs are specifically exposed to airborne microbes, which may explain the differences in the spleen samples. Although the tonsils are also exposed to airborne microbes, there was little similarity in the differential taxa abundances between the lungs and tonsils. This shows that the oral uptake of microbes was more influential for the tonsil microbiome composition. Nevertheless, *Anoxybacillus* and *Mycoplasma* were abundant in the lung, blood, and spleen samples of certain pigs in the present study. Both genera are found as inhabitants of the swine respiratory tract [31], which may be the origin of these taxa in the blood. *Mycoplasma* not only causes infectious anaemia in pigs, but it is also an important zoonotic agent for humans [32]. Another dominant taxon in four lung samples was *Anoxybacillus*, which is a genus belonging to the common lung microbiome of healthy pigs [31] but also represents food spoilage bacteria [33]. Interestingly, the genus *Ralstonia* was characteristic in nine spleen samples. *Ralstonia* species are plant pathogens [34]. Therefore, their presence in gut samples, especially in the cecal mucosa, was not surprising. However, their detection in the spleen samples may indicate bacterial transfer from the gut to the spleen, as previously reported [33,35]. Further genera that were present in the communities in the lung and spleen samples comprise spoilage bacteria and pathogens that were also found in the meat (i.e., *Acinetobacter*, *Escherichia*, *Pseudomonas*, *Streptococcus*, and *Campylobacter*).

Although the present results confirm the usefulness of utilizing 16S amplicon sequencing to monitor the microbiome composition of pig meat, blood, and visceral organs, this approach has limitations. A limitation is that the 16S rRNA region does not contain sufficient sequence variability to distinguish species or strains (e.g., a pathogenic vs. a commensal strain of *Escherichia coli*) [36]. Moreover, 16S rRNA gene sequencing cannot be used to describe the virulence of taxa. Therefore, the metagenomics or quantitative PCR of virulence genes should be included in future studies. Lastly, the 16S rRNA gene copies are obtained from total extracted DNA, which includes living and dead cells, and is only a semi-quantitative approach [36]. Accordingly, culture- or RNA-based methods for the detection of important foodborne pathogens within genera, such as *Campylobacter*, *Salmonella,* and *Escherichia coli*, targeting exclusively viable bacteria, should also be implemented in future studies. As the library preparation for certain samples was not successful, DNA extraction protocols should be optimized for a low DNA load. Nevertheless, the present 16S rRNA amplicon data provide useful information for implementing targeted bacterial monitoring approaches (e.g., quantitative PCR) at slaughterhouses.

## 5. Conclusions

The present results show the usefulness of 16S amplicon sequencing to monitor the microbiome composition of pork products and edible organs. However, it should be considered that the present study was conducted at a single meat processing plant and with a limited number of pigs from one farm. Therefore, the results presented here may be specific to this pilot study. Nevertheless, the present results emphasize the importance of monitoring bacterial ecology in edible organs, such as the spleen, lungs, and tonsils, as risk factors for their raw consumption and possible sources for the contamination of meat products. The observed batch-to-batch and animal-to-animal variation in the bacterial communities in the meat, blood, and organ samples is useful information to be considered for targeted interventions to minimize bacterial contamination during carcass processing, upstream packaging, and cooling of the finished product. Despite the fact that our findings were made at a single slaughterhouse, the present data demonstrate the importance of implementing similar monitoring measures in small- and medium-sized slaughterhouses. Moreover, our results provide useful information for targeted intervention strategies to enhance hygiene measures at slaughterhouses.

## Figures and Tables

**Table 1:** Alpha-diversity indices in the bacterial community among the sample types.

Sample Type	Index	SEM
	Observed features	
Feces	551 ^ab^	45.2
Cecal digesta	393 ^bc^	43.0
Cecal mucosa	635 ^a^	43.0
Blood	227 ^c^	47.6
Tonsil	370 ^bc^	43.0
Lung	284 ^bc^	50.5
Spleen	493 ^ab^	45.2
Meat cut	381 ^bc^	53.9
Hand (swab sample)	249 ^bc^	62.9
*p*-value	<0.001	
	Shannon	
Feces	4.67 ^a^	0.237
Cecal digesta	5.08 ^a^	0.225
Cecal mucosa	5.48 ^a^	0.225
Blood	3.08 ^b^	0.249
Tonsil	3.93 ^ab^	0.225
Lung	2.80 ^bc^	0.264
Spleen	2.72 ^c^	0.237
Meat cut	4.07 ^ab^	0.282
Hand (swab sample)	3.58 ^bc^	0.330
*p*-value	<0.001	
	Simpson	
Feces	0.949 ^a^	0.032
Cecal digesta	0.980 ^a^	0.030
Cecal mucosa	0.986 ^a^	0.030
Blood	0.758 ^b^	0.033
Tonsil	0.927 ^a^	0.030
Lung	0.745 ^b^	0.035
Spleen	0.695 ^b^	0.032
Meat cut	0.922 ^ab^	0.038
Hand (Swab sample)	0.912 ^ab^	0.044
*p*-value	<0.001	

The values are least-squares means ± standard error of the mean (SEM). The sample types were feces, cecal mucosa and digesta, blood, edible pork organs (i.e., lungs, tonsils, and spleen), meat, and swab samples from the hands of the workers at the slaughterhouse. The swabs were incubated in nutrient broth for 48 h before DNA extraction. The a,b,c superscript indicates a significant difference in the diversity index in the column (ANOVA and Tukey’s HSD test, *p* < 0.001).

## Data Availability

The raw sequences were deposited into the NCBI Bioproject databank under accession number PRJNA1124227.

## Supplementary Materials

The following supporting information can be downloaded at: https://www.mdpi.com/article/10.3390/pathogens14050475/s1, Table S1: Permutational Multivariate Analysis of Variance (PERMANOVA); Table S2: Selected bacterial genera (50 most abundant genera, % of all reads) in all sample types.
