# Supplementary material for: The Microbiome Characterization of Edible Visceral Organs and Fresh Meat During Production in a Pig Processing Facility in Thailand
Source: Pathogens. 2025 May 14;14(5):475. doi: 10.3390/pathogens14050475 (PMC12114174; doi:10.3390/pathogens14050475)
Supplement: Supplementary file 1 [file pathogens-14-00475-s001.zip › pathogens-3625540-supplementary.pdf]

pathogens

MDPI

Article

The Microbiome Characterization of Edible Visceral Organs and Fresh Meat During Production in a Pig Processing Facility in Thailand

Jutamat Klinsoda [1], Alongkot Boonsoongnern [2], Narut Thanantong [2], Tanyanant Kaminsonsakul [2], Khemmapas Treesuwan [1], Sudsai Trevanich [3] and Barbara U. Metzler-Zebeli [4,*]

1 Institute of Food Research and Product Development, University of Kasetsart, Bangkok 10900, Thailand; ifrjmk@ku.ac.th (J.K.); khemmapas.tr@ku.th (K.T.)
2 Department of Farm Resources and Production Medicine, Faculty of Veterinary Medicine, University of Kasetsart Kamphaeng Saen, Nakhon Pathom 73140, Thailand; fvetakb@ku.ac.th (A.B.); narut.t@ku.th (N.T.); tanyanant.k@ku.th (T.K.)
3 Department of Food Science and Technology, Faculty of Agro-Industry, University of Kasetsart, Bangkok 10900, Thailand; sudsai.t@ku.th
4 Centre for Veterinary Systems Transformation and Sustainability, Clinical Department for Farm Animals and Food System Science, University of Veterinary Medicine Vienna, 1210 Vienna, Austria
* Correspondence: barbara.metzler@vetmeduni.ac.at

Supplementary File

Table S1. Permutational Multivariate Analysis of Variance (PERMANOVA).

Source of Variation		df	Sums of Square	R2	Pseudo-F	P(perm)
Sample type [1]		8	13.79	0.42	7.88	0.001 ***
	Residuals	84	18.37	0.57		
	Total	92	32.17	1.00		
Location [2]		1	1.10	0.03	3.23	0.001 ***
	Residuals	91	31.07	0.96		
	Total	92	32.17	1.00		
Gut site [3]		2	1.40	0.21	4.48	0.001 ***
	Residuals	32	5.00	0.78		
	Total	34	6.40	1.00		

P(perm) mean p-perm value for a node permutation test. *** Significant code at P < 0.001.

[1] Sample types indicated the groups of samples: feces, blood, pork organs (i.e. lung, tonsil, spleen, and meat cut), and gut site (i.e. cecal mucosa and cecal digesta) and swab test from the hands of the staff at the slaughterhouse.

[2] Location of sampling: at the farm or at slaughterhouse.

[3] Gut site: cecal mucosa, cecal digesta and feces.

 https://doi.org/10.3390/pathogens14050475

Table S2. Selected bacterial genera (50 most abundant genera, % of all reads) in all sample types.

**Genus**	**Sample Type**										***P*-value**		
	F	B	DC	CM	H	L	M	S	T	SEM	Sample type	Pig	Replicate
UCG_005	10.0	2.9	27.5	12.8	2.8	0.8	1.1	1.3	1.1	0.145	<0.001***	0.256	0.971
Acinetobacter	0.1	1.2	0.6	0.5	15.3	16.0	35.9	6.0	1.6	0.188	<0.001***	0.915	0.172
Anoxybacillus	0.1	32.0	0.0	0.0	0.0	22.3	0.2	1.1	0.0	0.124	<0.001***	0.005**	0.918
Ralstonia	0.4	0.7	0.1	0.7	1.1	0.8	0.3	38.1	1.4	0.210	<0.001***	0.437	0.480
Escherichia_Shigella	2.9	0.5	0.9	1.3	9.5	0.6	14.0	2.0	9.1	0.160	<0.001***	0.769	0.606
Pseudomonas	0.3	2.6	0.3	0.5	17.5	6.9	2.3	1.9	0.5	0.150	<0.001***	0.112	0.603
Streptococcus	3.4	0.4	1.6	0.7	0.9	0.6	7.6	2.4	8.1	0.160	<0.001***	0.460	1.000
Christensenellaceae _R_7_group	9.7	1.6	3.8	2.9	0.4	0.4	0.5	0.9	0.5	0.140	<0.001***	0.191	0.917
Campylobacter	9.9	0.1	2.1	3.8	0.2	0.1	1.6	0.4	1.7	0.210	0.252	0.293	0.625
Mycoplasma	0.0	12.1	0.0	0.0	0.0	5.4	0.0	3.9	0.1	0.200	0.765	0.257	0.460
Fusobacterium	0.1	0.0	0.1	0.1	0.0	0.0	1.7	0.5	15.9	0.180	<0.001***	0.196	0.318
*Clostridium_* sensu_stricto_1	1.9	0.7	3.8	5.8	1.2	0.9	2.7	1.5	0.9	0.127	<0.001***	0.639	0.767
Bacteroides	1.3	1.5	0.5	1.6	0.2	0.8	4.2	1.5	5.4	0.190	0.025 *	0.310	0.087
Porphyromonas	0.0	0.0	0.0	0.1	0.0	0.2	0.6	0.3	13.2	0.170	<0.001***	0.773	1.000
Aeromonas	0.1	0.0	0.1	0.1	17.9	1.7	0.3	0.1	0.1	0.100	<0.001***	0.148	0.837
Helicobacter	7.2	0.0	0.2	3.9	0.3	0.1	0.9	0.9	0.8	0.190	0.020 *	0.229	0.842
UCG_002	6.5	1.2	2.8	1.6	0.3	0.3	0.4	0.8	0.4	0.110	0.0003 ***	<0.001***	0.927
Prevotella	0.4	0.1	4.6	4.6	0.4	1.3	0.8	0.9	0.4	0.150	<0.001***	0.466	0.942
Klebsiella	0.5	0.1	0.2	3.0	5.0	0.1	0.6	0.1	4.0	0.160	0.008 **	0.032 *	0.550
Methanobrevibacter	7.2	2.8	1.2	0.6	0.0	0.0	0.2	0.2	0.0	0.190	0.111	0.027 *	0.429
*NK4A214*_group	3.6	0.9	2.5	1.4	0.3	0.3	0.3	0.5	0.2	0.090	<0.001***	0.0003 ***	0.887

Lactobacillus	0.4	1.4	1.3	3.6	0.3	1.2	0.3	0.9	0.2	0.150	0.258	0.031 *	0.992
Ruminococcus	1.8	0.2	2.9	1.5	0.4	0.4	0.2	0.7	0.5	0.130	<0.001***	0.347	0.520
Actinobacillus	0.1	0.0	0.0	0.0	0.0	0.0	0.0	0.1	7.6	0.150	0.253	0.510	0.707
Romboutsia	0.2	0.2	0.7	1.0	0.4	4.9	0.3	0.7	0.5	0.100	<0.001***	0.458	0.967
*Prevotellaceae_* NK3B31_group	0.3	0.1	2.6	3.2	0.1	0.1	0.2	0.2	0.1	0.110	0.0001***	0.896	0.530
*Lachnospiraceae_* XPB1014_group	1.2	0.1	2.6	1.8	0.6	0.0	0.3	0.2	0.2	0.110	0.0004***	0.072	0.998
Alloprevotella	0.1	1.2	1.2	2.6	0.0	0.7	0.2	0.1	0.3	0.150	0.0001***	0.800	0.618
Prevotella_9	0.0	0.0	0.4	0.7	0.3	4.3	0.8	0.3	0.2	0.140	0.069	0.913	0.646
Faecalibacterium	1.5	0.0	0.5	0.3	0.3	2.2	0.5	0.5	0.5	0.160	0.171	0.566	0.881
*Rikenellaceae_* RC9_gut_group	1.3	0.1	1.2	1.9	0.2	0.2	0.2	0.4	0.1	0.150	0.003 **	0.190	0.096
Subdoligranulum	3.7	0.0	0.6	0.5	0.1	0.2	0.1	0.3	0.2	0.150	0.888	0.111	0.770
Succinivibrio	0.7	0.0	2.0	1.9	0.3	0.1	0.4	0.1	0.1	0.170	0.829	0.834	0.938
Ligilactobacillus	0.0	3.4	0.1	0.1	0.0	1.5	0.2	0.7	0.1	0.150	0.31	0.411	0.911
Agathobacter	0.6	0.1	1.6	1.5	0.4	0.2	0.2	0.4	0.2	0.080	0.006 **	0.390	0.807
Terrisporobacter	0.5	0.1	1.3	1.9	0.3	0.3	0.2	0.2	0.1	0.110	0.026 *	0.544	0.359
Bifidobacterium	0.9	0.1	0.0	0.3	0.4	0.5	1.0	1.0	0.7	0.200	0.334	0.350	0.189
Monoglobus	0.9	0.2	1.7	1.2	0.2	0.0	0.1	0.1	0.0	0.130	0.239	0.029 *	0.394
Roseburia	0.5	0.1	1.5	1.2	0.3	0.3	0.2	0.1	0.3	0.100	0.139	0.959	0.501
Anaerovibrio	0.1	0.1	0.4	3.3	0.1	0.0	0.0	0.1	0.0	0.140	<0.001***	0.173	0.013 *
Moraxella	0.0	0.2	0.0	0.0	0.0	0.9	2.8	0.9	0.4	0.180	0.679	0.449	0.912
Oscillospira	1.0	0.1	1.3	1.4	0.2	0.0	0.1	0.1	0.0	0.090	0.013 *	0.512	0.624
Treponema	0.7	0.0	1.0	1.0	0.1	0.0	0.3	0.1	0.6	0.190	0.648	0.657	0.168
Dysgonomonas	0.0	0.0	0.1	0.0	5.0	0.0	0.0	0.0	0.0	0.130	<0,001 ***	0.710	0.695
Oscillibacter	0.9	0.4	0.9	0.7	0.2	0.1	0.2	0.2	0.1	0.110	0.029 *	0.716	0.368
Phascolarctobacterium	0.4	0.1	0.7	1.8	0.1	0.1	0.1	0.2	0.1	0.130	<0.001***	0.491	0.024 *
Blautia	0.3	0.2	0.5	0.4	0.2	0.2	0.4	0.9	0.4	0.120	0.001**	0.229	0.323

Peptostreptococcus	0.0	0.0	0.1	0.2	0.2	0.3	0.2	0.2	2.1	0.150	0.013 *	0.532	0.513
*Prevotellaceae_ UCG_*003	0.1	0.1	1.2	1.2	0.1	0.0	0.1	0	0.0	0.140	0.013 *	0.041 *	0.935
Parabacteroides	0.4	0.1	0.5	1.3	0.0	0	0.2	0.1	0.1	0.110	0.012 *	0.463	0.977

Values are least-squares means of relative abundance ± standard error of the mean (SEM). B = blood, CM = cecal mucosa, DC = cecal digesta, F = feces, H = carcass handlers' hand, L = lung, M = meat, S = spleen, T = tonsil. * Significant code at $P < 0.05$ ** Significant code at $P < 0.01$ *** Significant code at $P < 0.001$.
